# D‐mannose alleviates osteoarthritis progression by inhibiting chondrocyte ferroptosis in a HIF‐2α‐dependent manner

**DOI:** 10.1111/cpr.13134

**Published:** 2021-09-25

**Authors:** Xueman Zhou, Yingcheng Zheng, Wentian Sun, Zhenzhen Zhang, Jiaqi Liu, Wenke Yang, Wenxiu Yuan, Yating Yi, Jun Wang, Jin Liu

**Affiliations:** ^1^ State Key Laboratory of Oral Diseases and National Clinical Research Center for Oral Diseases Department of Orthodontics West China Hospital of Stomatology Sichuan University Chengdu China; ^2^ Lab for Aging Research State Key Laboratory of Biotherapy and National Clinical Research Center for Geriatrics West China Hospital Sichuan University Chengdu China

## Abstract

**Objectives:**

Chondrocyte ferroptosis contributes to osteoarthritis (OA) progression, and D‐mannose shows therapeutic value in many inflammatory conditions. Here, we investigated whether D‐mannose interferes in chondrocyte ferroptotic cell death during osteoarthritic cartilage degeneration.

**Materials and methods:**

In vivo anterior cruciate ligament transection (ACLT)‐induced OA mouse model and an in vitro study of chondrocytes in an OA microenvironment induced by interleukin‐1β (IL‐1β) exposure were employed. Combined with *Epas1* gene gain‐ and loss‐of‐function, histology, immunofluorescence, quantitative RT‐PCR, Western blot, cell viability and flow cytometry experiments were performed to evaluate the chondroprotective effects of D‐mannose in OA progression and the role of hypoxia‐inducible factor 2 alpha (HIF‐2 α) in D‐mannose‐induced ferroptosis resistance of chondrocytes.

**Results:**

D‐mannose exerted a chondroprotective effect by attenuating the sensitivity of chondrocytes to ferroptosis and alleviated OA progression. HIF‐2α was identified as a central mediator in D‐mannose‐induced ferroptosis resistance of chondrocytes. Furthermore, overexpression of HIF‐2α in chondrocytes by Ad‐*Epas1* intra‐articular injection abolished the chondroprotective effect of D‐mannose during OA progression and eliminated the role of D‐mannose as a ferroptosis suppressor.

**Conclusions:**

D‐mannose alleviates osteoarthritis progression by suppressing HIF‐2α‐mediated chondrocyte sensitivity to ferroptosis, indicating D‐mannose to be a potential therapeutic strategy for ferroptosis‐related diseases.

## Graphical abstract

D‐Mannose effectively alleviates osteoarthritis progression by suppressing cartilage degeneration. D‐mannose protects osteoarthritic chondrocytes by attenuating sensitivity to ferroptosis. HIF‐2α is vital in potentiating the susceptibility of chondrocytes to ferroptosis. HIF‐2α is a central mediator in D‐mannose‐induced ferroptosis resistance.

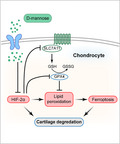

## 1 INTRODUCTION

Ferroptosis is a form of oxidative cell death characterized by the iron‐dependent accumulation of lipid hydroperoxides to lethal levels.^1^, ^2^, ^3^ Ferroptosis has emerged as a potent mechanism for preventing multiple neoplastic and degenerative diseases such as Alzheimer's disease, Parkinson's disease and kidney degeneration.^1^, ^4^ As one of the most common degenerative joint disorders, osteoarthritis (OA) is the leading cause of chronic disability in the geriatric population, imposing a huge burden on society.^5^ Current medical care for OA focuses mainly on alleviating painful symptoms, but typically fails to prevent disease progression. Pathologically, degradation of articular cartilage is one of the most prominent features of progressive OA.^6^ Articular cartilage is composed of chondrocytes, which maintain the integrity of the extracellular matrix by balancing its synthesis and degradation.^7^ Evidence shows that anabolic/catabolic balance and survival of chondrocytes are crucial for articular cartilage homeostasis and osteoarthritic destruction,^8^ underlining the importance of chondrocyte fate control. Recent evidence has indicated that chondrocyte ferroptosis contributes to the progression of OA.^9^ Iron‐overloaded mice exhibit increased cartilage destruction, and intracellular iron uptake is favoured in chondrocytes mimicking an OA phenotype.^10^, ^11^ These findings suggest that ferroptosis suppression is a novel candidate component for preventing OA progression.

D‐mannose, a C‐2 epimer of glucose, is naturally present in many fruits and plants, and has been reported to be beneficial in many human disease states.^12^ For example, it has been shown to be effective in tumour inhibition, immunopathology repression and infection treatment through anti‐inflammatory and immunomodulatory activity.^13^, ^14^, ^15^ Lin et al.^16^ reported that D‐mannose could suppress monosodium iodoacetate‐induced OA development in rats, and it was reported that D‐mannose inhibits LPS‐induced production of IL‐1β, which also mediates OA progression.^17^ Evidence has verified the roles of D‐mannose in glucose metabolism, anti‐inflammation and T‐cell immune response.^13^, ^17^, ^18^, ^19^ To bring the role of chondrocyte ferroptosis in OA into focus, the relationship between D‐mannose and chondrocyte ferroptosis needs elucidation.

Hypoxia‐inducible factor 2α (HIF‐2α) has been reported to play a vital role in cartilage development, OA progression and sensitizing cells to ferroptosis.^20^, ^21^, ^22^ In embryonic development, HIF‐2α insufficiency impairs not only chondrocyte hypertrophy but also the subsequent steps of endochondral ossification.^23^ During OA, HIF‐2α performs as a key catabolic transcription factor, inducing the expression of matrix‐degrading enzymes and progressive cartilage damage under the regulation of NF‐κB signalling.^23^, ^24^, ^25^, ^26^ Recently, work focussing on tumours has demonstrated an essential role for HIF‐2α in regulating cellular iron homeostasis and ferroptosis susceptibility.^21^, ^22^ For example, HIF‐2α was shown to stimulate the specific enrichment of polyunsaturated fatty acids in clear cell renal cell carcinoma,^27^ whereas in colorectal cancers, HIF‐2α activation potentiates oxidative cell death by increasing cellular iron.^21^ Until now, it has remained unknown whether HIF‐2α contributes to chondrocyte ferroptosis and its regulation in OA and how it is regulated.

Here, we employed an anterior cruciate ligament transection (ACLT)‐induced OA mouse model and an in vitro OA microenvironment induced by IL‐1β exposure. Our results demonstrated that D‐mannose possesses chondroprotective effects against OA progression through inhibiting HIF‐2α‐potentiated chondrocyte ferroptosis. These findings not only provide valid evidence for D‐mannose to be used in clinical interventions in OA, but also suggest the potential therapeutic prospects of the natural plant‐derived component for many other ferroptosis‐related diseases.

## 2 MATERIALS AND METHODS

### 2.1 Mice and surgery

C57BL/6 J mice (8 weeks old, female) were purchased from Dossy Experimental Animal Limited Company (Chengdu, China). All the mice were bred and maintained under specific‐pathogen‐free conditions on a 12‐/12‐h light/dark cycle. For D‐mannose (Sigma‐Aldrich, M2069) treatment, normal drinking water was exchanged for 20% mannose in drinking water (w/v) according to previous studies.^13^, ^14^ For surgery, mice were anaesthetized with pentobarbital sodium (100 mg/kg, injected intraperitoneally) and subjected to unilateral ACLT procedures.^28^ The sham group received a skin incision and suturing without patellar dislocation or ligament transection. For virus injection, mice were intraarticularly injected with 1 × 10^9^ pfu (8 µl) of mock or Ad‐*Epas1* virus after one week of surgery. For Fer‐1 (MCE, Monmouth Junction, HY‐100579) injection, mice were intraarticularly injected with 1 mg/kg Fer‐1 or with vehicle two weeks after surgery, the injection was repeated once a week. Mice were sacrificed 4 and 8 weeks after surgery for analyses. The animal protocol was approved by Subcommittee on Research and Animal Care (SRAC) of Sichuan University (No. WCHSIRB‐D‐2019‐092).

### 2.2 Histology and immunofluorescence

After fixation, mouse legs were decalcified in 0.5 M EDTA for 2 weeks, embedded in paraffin and sectioned at 4 μm. Slides were stained with safranin O/fast green (Solarbio) using standard protocol. The severity of OA‐like phenotype was analysed using the OARSI scoring system by two blinded observers.

For immunofluorescence analysis, after antigen retrieval and blocking, sections or fixed chondrocytes were incubated at 4°C overnight with primary antibodies against COL2A1 (Abcam, ab34712), MMP13 (Abcam, ab39012), HIF‐2α (Novus, NB100‐132), GPX4 (Abcam, ab125066), NF‐κB p65(sc‐8008) and p‐NF‐κB p65 (Cell Signaling Technology, #3003). The secondary antibodies included donkey anti‐mouse Alexa Fluor 488/555 and donkey anti‐rabbit Alexa Fluor 488/555 (all from Thermo Fisher Scientific). The nucleus was counterstained using 4′,6‐diamidino‐2‐phenylindole (DAPI; Sigma, D9542). Images were acquired with a Nicon A1 confocal microscope and processed and analysed with ImageJ software.

### 2.3 Isolation and culture of mouse chondrocytes

Primary mouse chondrocytes were isolated from knee joint cartilage of 5‐day‐old C57BL/6 J mice as described previously.^29^ Briefly, after dissected into pieces, cartilage tissue was digested by 2.5mg/ml collagenase type II (Gibco) for 2 h and 0.5 mg/ml collagenase type II overnight at 37°C. The primary chondrocytes were resuspended and cultured in low glucose DMEM medium (Gibco) containing 10% foetal bovine serum (Gibco) and 1% penicillin‐streptomycin at 37°C with an atmosphere of 21% O_2_ (for normoxia) or 1% O_2_ (for hypoxia), 5% CO_2_ and 95% humidity.

Primary chondrocytes were identified with toluidine blue (Solarbio), safranin O (Solarbio) staining and COL2A1 immunofluorescence according to manufacturer instructions. To guarantee the phenotype integrity, we only use first‐passage chondrocytes. Primary chondrocytes were incubated with recombinant murine IL‐1β (10 ng/ml, Peprotech, 211‐11B), D‐mannose (25 mM, Sigma, M2069), erastin (10 µM, MCE, HF‐15763), Fer‐1 (1 µM, MCE, HY‐100579) and etomoxir (20 µM, MCE, HY‐50202).

### 2.4 Small interfering RNA assays

siRNAs specific to *Epas1* was designed with the coding sequences of mouse *Epas1* shown in Table S1. Chondrocytes cultured for 3 days were transfected for 24 h with siRNA (100 nM) using Lipofectamine 3000 (Invitrogen). Non‐silencing siRNA was used as a negative control.

### 2.5 Epas1 adenovirus and infection

Adenovirus expressing mouse *Epas1* and mock virus were produced from Genechem. For infection of primary chondrocytes, chondrocytes were cultured for 3 days, infected with 800 MOI of mock virus or Ad‐*Epas1* virus for 12 h, and incubated for additional 36 h.

### 2.6 CCK‐8 cell viability assay

Primary chondrocytes were transferred to 96‐well plates at a concentration of 5000 cells/well in 100 μl of culture medium supplemented with 10 μl of CCK‐8 reagent (MCE) and incubated at 37°C for 2 h following indicated treatments. Cell viability was evaluated using the absorbance values determined at 450 nm using microplate reader (Synergy H1; BioTek).

### 2.7 Lipid peroxidation, ROS assay

To detect lipid peroxidation, cells were incubated with 5 µM of BODIPY581/591 C11 (Invitrogen, D3861) for 45 min, washed with PBS twice, trypsinized and filtered into single‐cell suspensions. Flow cytometry analysis was performed on BD FACS Aria II (Becton Dickinson), using the PE‐TexasRed filter for reduced BODIPY‐C11 (emission: 590 nm) and the FITC filter for oxidized BODIPY‐C11 (emission: 510 nm). A minimum of 20,000 cells were analysed for each sample. Data analysis was performed using the FlowJo v10 (BD Bioscience). DCFH‐DA (Beyotime, S0033) staining was used to measure ROS and assayed by fluorescence microscopy (Ts2R/FL; Nicon).

### 2.8 MDA, GSH and SOD assay

Primary chondrocytes or cartilage tissue was used to determine malonaldehyde (MDA) concentration, glutathione (GSH) content and total superoxide dismutase (SOD) activity in accordance with manufacturers’ protocols with the following kits: Lipid Peroxidation MDA Assay Kit (Beyotime, S0131), GSH and GSSG Assay Kit (Beyotime, S0053) and Total Superoxide Dismutase Assay Kit with WST‐8 (Beyotime, S0101).

### 2.9 Mitochondrial and lipid droplet staining

For mitochondrial staining, cells were labelled with MitoTracker Red (50 nM, Beyotime, C1035) at 37°C for 30 min, then washed with PBS and fixed with 4% paraformaldehyde for 15 min. For lipid droplet staining, fixed cells were stained for 30 minutes with 0.1 µg/ml Nile Red (MCE, HY‐D0718), and the nucleus was counterstained using DAPI (Sigma). Images were acquired with a Nicon A1 confocal microscope and processed and analysed with ImageJ software.

### 2.10 Total RNA extraction and quantitative real‐time PCR analysis

Total RNA of cartilage tissue/cultured chondrocytes was extracted using Animal Total RNA Isolation Kit/Cell Total RNA Isolation Kit (Foregene, RE‐03011/03111) according to the manufacturer's instructions. The RNA concentration was measured with a NanoDrop 2000 (Thermo Fisher Scientific), and cDNA was prepared using PrimeScript RT reagent Kit (Takara Bio Inc., RR047A). qRT‐PCR was performed using SYBR Premix Ex Taq II (Takara, RR420L) in CFX96 Real‐Time System (Bio‐Rad). Relative gene expression was normalized by *β*‐*actin* using the 2^−ΔΔCt^ method. The primers are listed in Table S2.

### 2.11 Western blotting

The cell lysates were extracted using RIPA lysis buffer (Beyotime, P0013B) containing 1 mM PMSF (Beyotime, ST505). The samples were heated at 95°C for 5 min in sample buffer containing 2% SDS and 1% mercaptoethanol, separated on 10% SDS‐polyacrylamide gels, and transferred to PVDF membranes by a wet transfer apparatus (Bio‐Rad). The membranes were blotted with 5% BSA for 1 h and then incubated at 4°C. Before incubation with the secondary antibodies, the membranes were washed in TBST solution three times. The following primary antibodies were used: MMP13 (Abcam, ab39012), HIF‐2α (Novus, NB100‐132), GPX4 (Abcam, ab125066), SLC7A11 (Abcam, ab175186), IκBα (Santa Cruz, sc‐1643), NF‐κB p65 (Santa Cruz, sc‐8008) and p‐NF‐κB p65 (Cell Signaling Technology, #3003) and β‐actin (Proteintech, 66009‐1‐Ig). The antibody‐antigen complexes were visualized with Immobilon reagents (Millipore, WBKLS0100).

### 2.12 Statistical analysis

Results are presented as the mean ± standard error of mean of independent replicates (*n* ≥ 3). Statistically significant differences were evaluated using two‐tailed Student's *t* tests for comparison between two groups or by one‐way analysis of variance followed by the Tukey's test for multiple comparisons. NEJM formatting for *p* values was used (NS when no significant difference, * when *p* < 0.05, ** when *p* < 0.01, *** when *p* < 0.001). All statistical analyses were conducted using GraphPad Prism 8.

## 3 RESULTS

### 3.1 D‐mannose alleviates OA progression and cartilage degeneration in a mouse ACLT model

**FIGURE 1:**
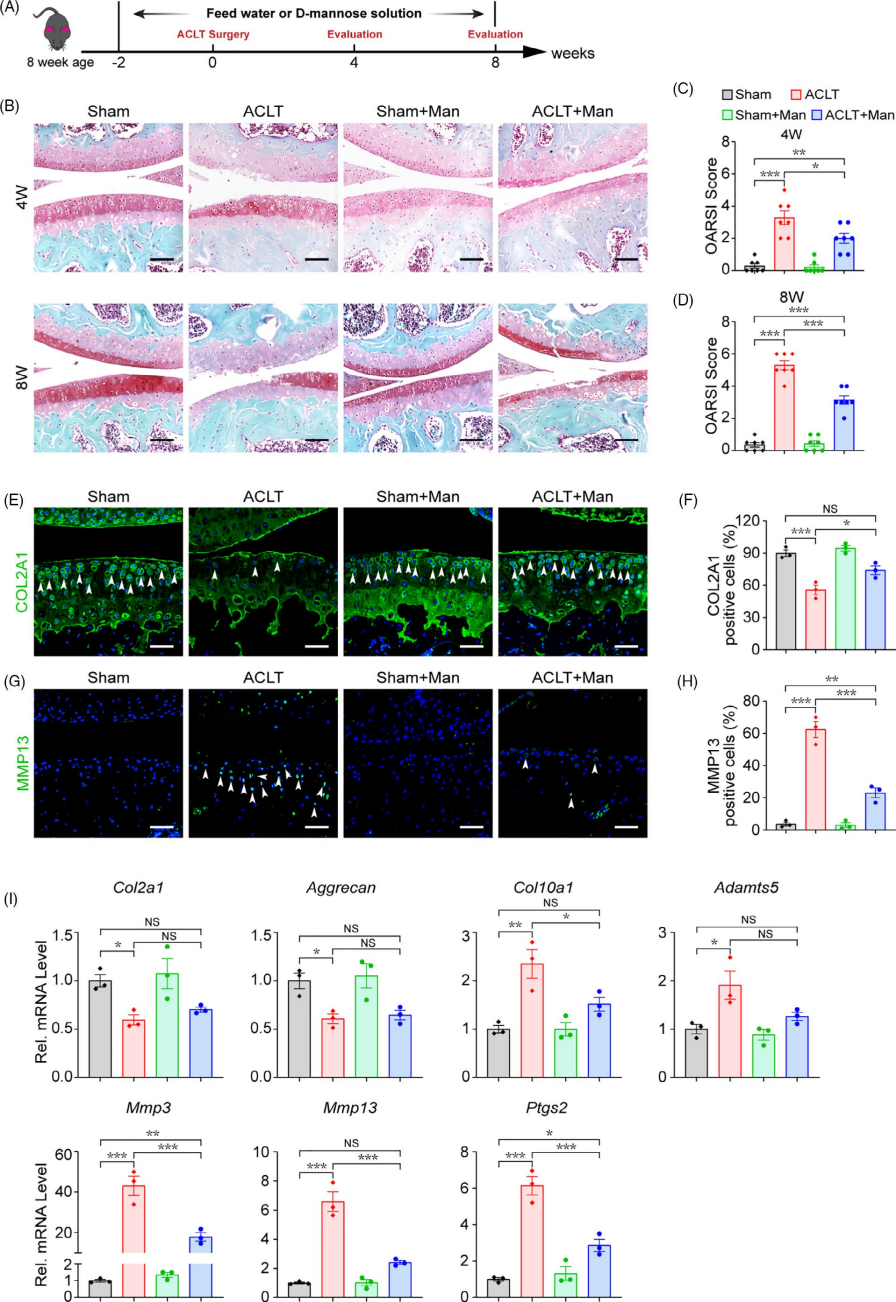
D‐mannose alleviates OA progression and cartilage degeneration in the mouse ACLT model. (A) Schematic model of the time course for establishment of the anterior cruciate ligament transection (ACLT) model of OA mouse treated with D‐mannose (Man) by administration in drinking water. (B) Representative safranin O/fast green staining of sham and ACLT‐induced OA mice treated with/without D‐mannose administration 4 or 8 weeks post‐surgery. Scale bars, 200 μm. (C and D) Osteoarthritis Research Society International (OARSI) score evaluated based on Safranin O/fast green staining (C) 4 or (D) 8 weeks post‐surgery. *n* = 7. (E and F) (E) Representative immunofluorescence staining of COL2A1 in knee joint 4 weeks post‐surgery and (F) quantification. Arrow heads indicated positive cells. *n* = 3. Scale bars, 100 μm. (G and F) (G) Representative immunofluorescence staining of MMP13 in knee joint 4 weeks post‐surgery and (H) quantification. Arrow heads indicated positive cells. *n* = 3. Scale bars, 100 μm. (I) Quantitative RT‐PCR analyses of the gene expression of knee joint cartilage tissues 4 weeks post‐surgery. *n* = 3. All quantified data are shown as mean ± SEM; NS, not significant, *p < 0.05, **p < 0.01, ***p < 0.001 by one‐way ANOVA followed by the Tukey‐Kramer test

To explore the role of D‐mannose in OA progression, we examined the dynamic changes in cartilage degeneration and the expression of related genes after ACLT surgery with (ACLT + Man group) or without (ACLT group) D‐mannose application. D‐mannose was added to the drinking water of mice beginning two weeks prior to ACLT surgery (Figure 1A). Safranine O staining of cartilage and Osteoarthritis Research Society International (OARSI) score analysis^30^ at 4 and 8 weeks post‐surgery showed that cartilage degeneration appeared soon after ACLT surgery and gradually increased in severity (Figure 1B–D). However, articular cartilage degradation was attenuated in the ACLT + Man group compared with the ACLT group, together with a significantly decreased OARSI grading score (Figure 1B–D). Immunofluorescence staining revealed that, compared to the ACLT group, the ACLT + Man group showed a significantly increased level of the matrix protein, collagen type II (COL2A1) (Figure 1E, F), and a significantly decreased level of the degradative enzyme, matrix metalloproteinase 13 (MMP13) (Figure 1G, H), in the cartilage. In addition, we examined the expression of genes related to cartilage degeneration and found that ACLT surgery resulted in decreased expression of anabolic factors such as *Col2a1* and *Aggrecan*, but increased expression of catabolic genes *Mmp3*, *Mmp13*, *Adamts5*, *Ptsg2*, as well as chondrocyte pathological hypertrophy marker, *Col10a1*, in the mouse articular cartilage. All of the expression changes described above were reversed by D‐mannose supplementation except for those of *Col2a1* and *Aggrecan* (Figure 1I). These results implied that D‐mannose alleviates OA progression mainly by suppressing articular cartilage degeneration.

### 3.2 **D‐mannose suppresses IL‐1β‐induced catabolism in chondrocytes**.

**FIGURE 2:**
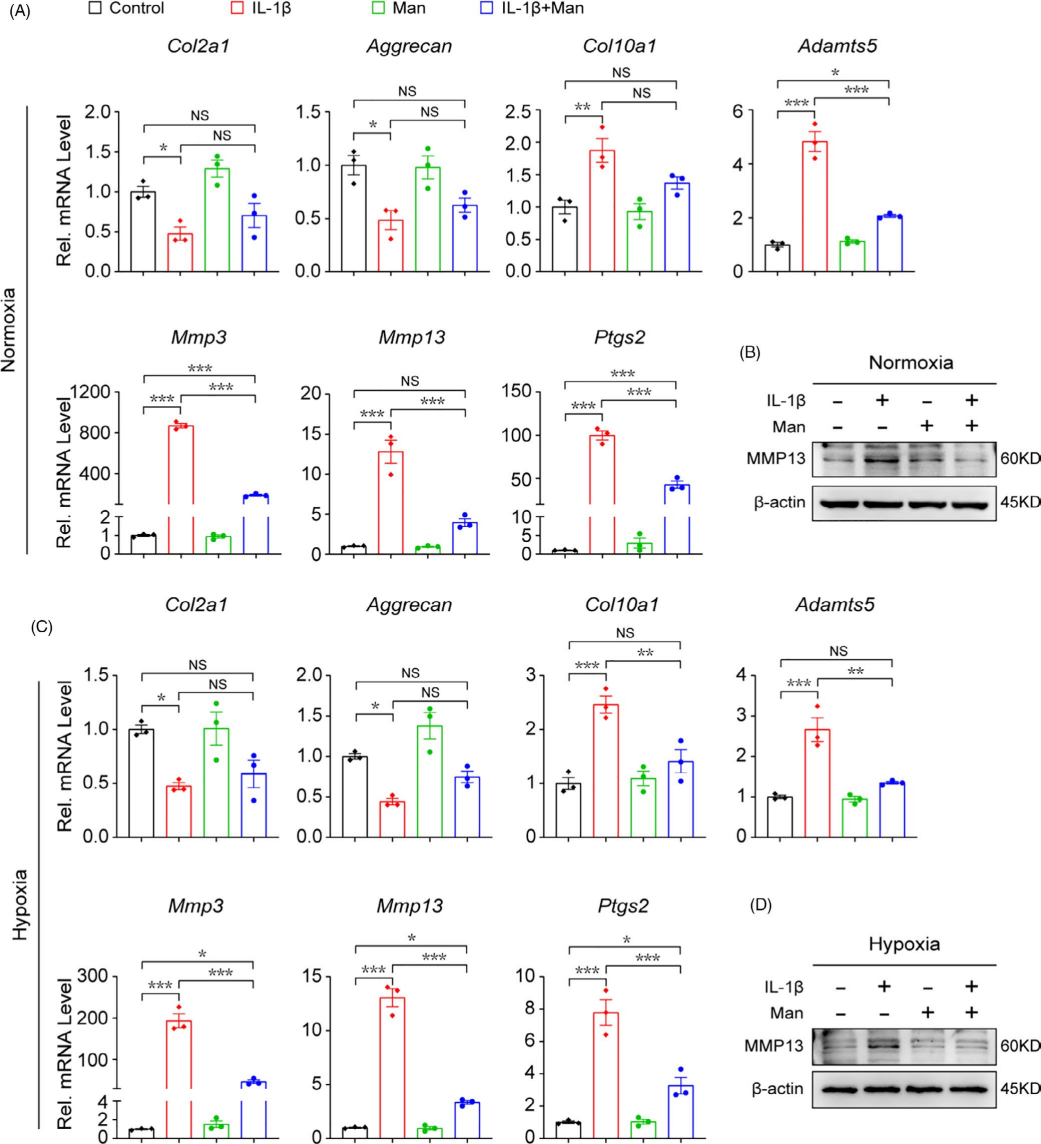
D‐mannose suppresses IL‐1β‐induced catabolism of chondrocyte. (A and B) (A) Quantitative RT‐PCR of the gene expression and (B) Western blot analyses of MMP13 of chondrocytes under normoxia cultured conditions 24 h post indicated treatment. *n* = 3. (C and D) (C) Quantitative RT‐PCR of the gene expression and (D) Western blot analyses of MMP13 of chondrocytes under hypoxia cultured conditions 24 h post indicated treatment. *n* = 3. Man, D‐mannose. All quantified data are shown as mean ± SEM; NS, not significant, *p < 0.05, **p < 0.01, ***p < 0.001 by one‐way ANOVA followed by the Tukey‐Kramer test

We next investigated the effect of D‐mannose on IL‐1β‐induced chondrocyte catabolism in vitro. We isolated primary chondrocytes from neonatal mice (Figure S1A) and incubated the cells in medium supplemented with D‐mannose or PBS in the presence of IL‐1β. Cell counting kit (CCK‐8) assay indicated that moderate concentrations (≤25 mM) of D‐mannose were non‐toxic to chondrocytes within our observation time (≤48 h) (Figure S1B). Consistent with in vivo data, the mRNA levels of catabolic genes (*Mmp3*, *Mmp13*, *Adamts5* and *Ptsg2*) and a chondrocyte pathological hypertrophy marker (*Col10a1*) in IL‐1β‐treated chondrocytes significantly decreased, but the mRNA levels of anabolic genes (*Col2a1* and *Aggrecan*) showed no change after D‐mannose administration. (Figure 2A). As articular cartilage is avascular, chondrocytes adapt to hypoxic conditions in vivo.^31^ So, we also cultured chondrocytes under hypoxic condition to mimic cartilage environment and to examine whether the effects of D‐mannose during normoxia are also exerted under hypoxic condition. As in normoxic conditions, upregulated expression of *Mmp3*, *Mmp13*, *Adamts5*, *Ptsg2* and *Col10a1* that were induced by IL‐1β significantly decreased after D‐mannose treatment (Figure 2C). Western blot analysis confirmed that D‐mannose effectively downregulated the MMP13 protein level (Figure 2B,D). These data confirmed that D‐mannose regulates cartilage homeostasis mainly by suppressing IL‐1β‐induced catabolism in chondrocytes.

### 3.3 D‐mannose protects osteoarthritic chondrocytes by attenuating sensitivity to ferroptosis

**FIGURE 3:**
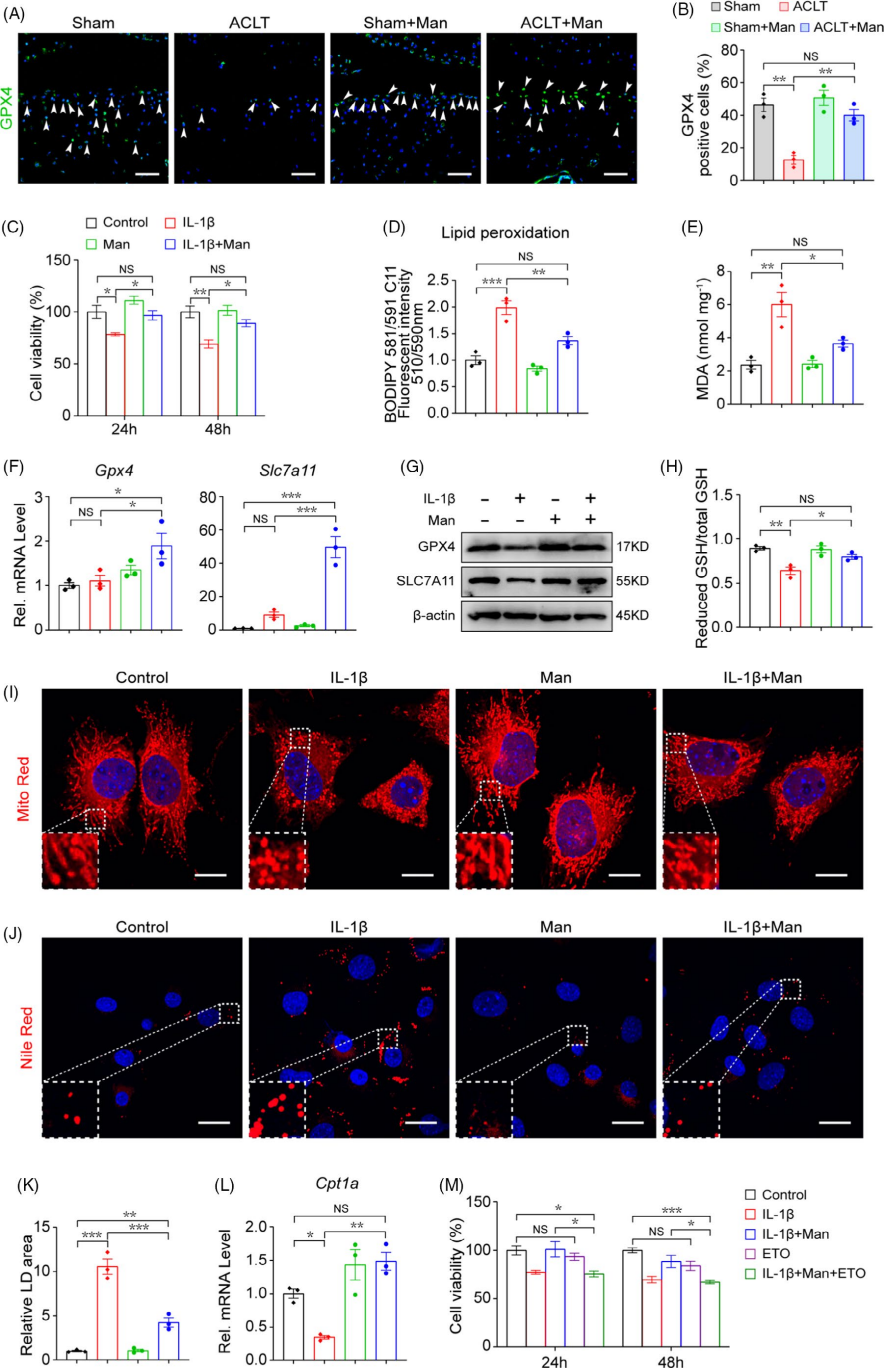
D‐mannose protects osteoarthritic chondrocytes by attenuating sensitivity to ferroptosis. (A) Representative immunofluorescence staining of GPX4 in knee joint 4 weeks post‐surgery and (B) quantification. Arrow heads indicated positive cells. *n* = 3. Scale bars, 100 μm. (C) Chondrocyte's cell viability determined by CCK‐8 assay 24 and 48 h post indicated treatment. *n* = 4. (D) Lipid peroxidation was determined using the BODIPY 581/591 C11 reagent in chondrocytes 24 h post indicated treatment. *n* = 3. (E) MDA measurement 24 h post indicated treatment. *n* = 3. (F and G) (F) Quantitative RT‐PCR and (G) Western blotting analyses of the Gpx4 and Slc7a11 of chondrocytes 24h post indicated treatments. *n* = 3. (H) Measurement of GSH content 24 h post indicated treatment. *n* = 3. (I) Representative images of chondrocytes 24 h post indicated treatments visualized with MitoTracker Red staining. Dotted areas were magnified in the left bottom. Scale bars, 20 μm. (J) Representative images of chondrocytes 24 h post indicated treatments stained with Nile Red to detect lipid droplets (LDs). Dotted areas were magnified in the left bottom. Scale bars, 40 μm. (K) Quantification of relative LDs area, *n* = 3. (L) Quantitative RT‐PCR analyses of the *Cpt1a* gene expression of chondrocytes 24 h post indicated treatments. *n* = 3. (M) Chondrocytes cell viability determined by CCK‐8 assay 24 and 48 h post indicated treatment. *n* = 4. Man, D‐mannose. All quantified data are shown as mean ± SEM; NS, not significant, *p < 0.05, **p < 0.01, ***p < 0.001 by one‐way ANOVA followed by the Tukey‐Kramer test

Recent evidence has indicated that chondrocyte ferroptosis contributes to chondrocyte homeostasis and osteoarthritic cartilage degeneration.^9^ Similar to previous study, ferrostatin‐1 (Fer‐1), the inhibitor of ferroptosis, could attenuate the cytotoxicity of IL‐1β to chondrocytes, indicating the existence of ferroptosis in IL‐1β‐treated chondrocytes (Figure S2A). GPX4 is a canonical glutathione‐based ferroptosis inhibitor and functions mainly through inhibiting the formation of lipid peroxides. To study the role of D‐mannose in chondrocyte ferroptosis, we first examined the percentage of GPX4^+^ chondrocytes in sham‐operated, sham‐operated D‐mannose treated (Sham + Man), ACLT and D‐mannose‐treated ACLT (Sham + Man) groups using immunohistochemistry staining assay. As expected, compared with the sham group, the percentage of GPX4^+^ chondrocytes in the ACLT group was reduced significantly (from 46.7 ± 9.1% to 12.5 ± 6.2%). To our surprise, we observed that D‐mannose treatment largely rescued ACLT‐induced GPX4^+^ chondrocyte reduction (Figure 3A,B). These in vivo results suggested that OA‐induced chondrocyte ferroptosis could be suppressed significantly by D‐mannose application.

To confirm the effect of D‐mannose on chondrocyte ferroptosis, we next investigated whether D‐mannose interfered in chondrocyte ferroptotic cell death during cartilage degeneration in vitro. As evidenced by cell viability assay, D‐mannose rendered the chondrocytes more resistant to IL‐1β‐potentiated cell death (Figure 3C). C11‐BODIPY staining showed that lipid peroxidation was significantly enhanced in response to IL‐1β stimulation and inhibited by D‐mannose (Figure 3D). Moreover, D‐mannose potently decreased the level of malondialdehyde (MDA), a by‐product of lipid peroxidation (Figure 3E). Also, the RNA and protein levels of the two key ferroptosis suppressors, Gpx4 and Slc7a11, were increased in D‐mannose‐treated chondrocytes compared with those treated with IL‐1β (Figure 3F,G). We tested reduced glutathione (GSH), which are essential for ferroptosis prevention,^27^ and found that reduced GSH level was downregulated in response to IL‐1β treatment and could be reversed by D‐mannose (Figure 3E). We also investigated the intracellular ROS level, which always increases significantly in ferroptosis. Expectedly, after D‐mannose treatment, we observed a much lower ROS level in control chondrocytes than in IL‐1β‐treated ones upon DCFH‐DA staining (Figure S3A,B). D‐mannose treatment also increased superoxide dismutase (SOD) gene expression and activity but decreased NAPDH oxidase 2 (*Nox2*) expression, both of which suggested an inhibited degree of oxidative stress (Figure S3C,D). MitoTracker Red staining revealed a striking ferroptosis‐associated morphologic change in chondrocytes upon treatment with IL‐1β, characterized by discontinuous, shrinking mitochondria. Notably, D‐mannose treatment successfully impeded IL‐1β‐induced mitochondrial impairment in chondrocytes (Figure 3I). Recently, ferroptosis has been demonstrated to be closely associated with lipid droplets (LDs), which can accumulate and sensitize cancer cells to ferroptosis.^27^, ^32^, ^33^ We stained LDs using Nile Red and found that the abundance of LDs was strongly reduced in IL‐1β+D‐mannose‐treated cells compared with IL‐1β‐treated ones (Figure 3J,K). β‐oxidation is involved in LD degeneration.^13^ Accordingly, expression of carnitine palmitoyl‐transferase 1A (*Cpt1a*), which encodes the enzyme responsible for β‐oxidation, was enhanced by D‐mannose treatment (Figure 3L) Furthermore, treatment with Etomoxir (ETO), an inhibitor of CPT1A, abolished D‐mannose‐enhanced chondrocyte viability (Figure 3M). Erastin can induce ferroptosis by activating voltage‐dependent anion channels and inhibiting the cystine‐glutamate antiporter system Xc‐.^34^ Consistent with IL‐1β treatment data, we also found that chondrocyte ferroptosis and catabolism induced by erastin were all significantly mitigated by D‐mannose application. D‐mannose rescued the erastin‐induced decrease in cell viability and abolished the erastin‐mediated augmentation of lipid peroxidation and mRNA levels of *Mmp3*, *Mmp13* and *Ptgs2* (Figure S4A–C).

Taken together, our results demonstrate that D‐mannose protects chondrocytes from ferroptosis mainly by preventing lipid storage and lipid peroxide accumulation. Moreover, we found that inhibition of CPT1A‐mediated β‐oxidation abolished D‐mannose‐enhanced chondrocyte viability.

### 3.4 D‐mannose alleviates cartilage degeneration by suppressing HIF‐2α

**FIGURE 4:**
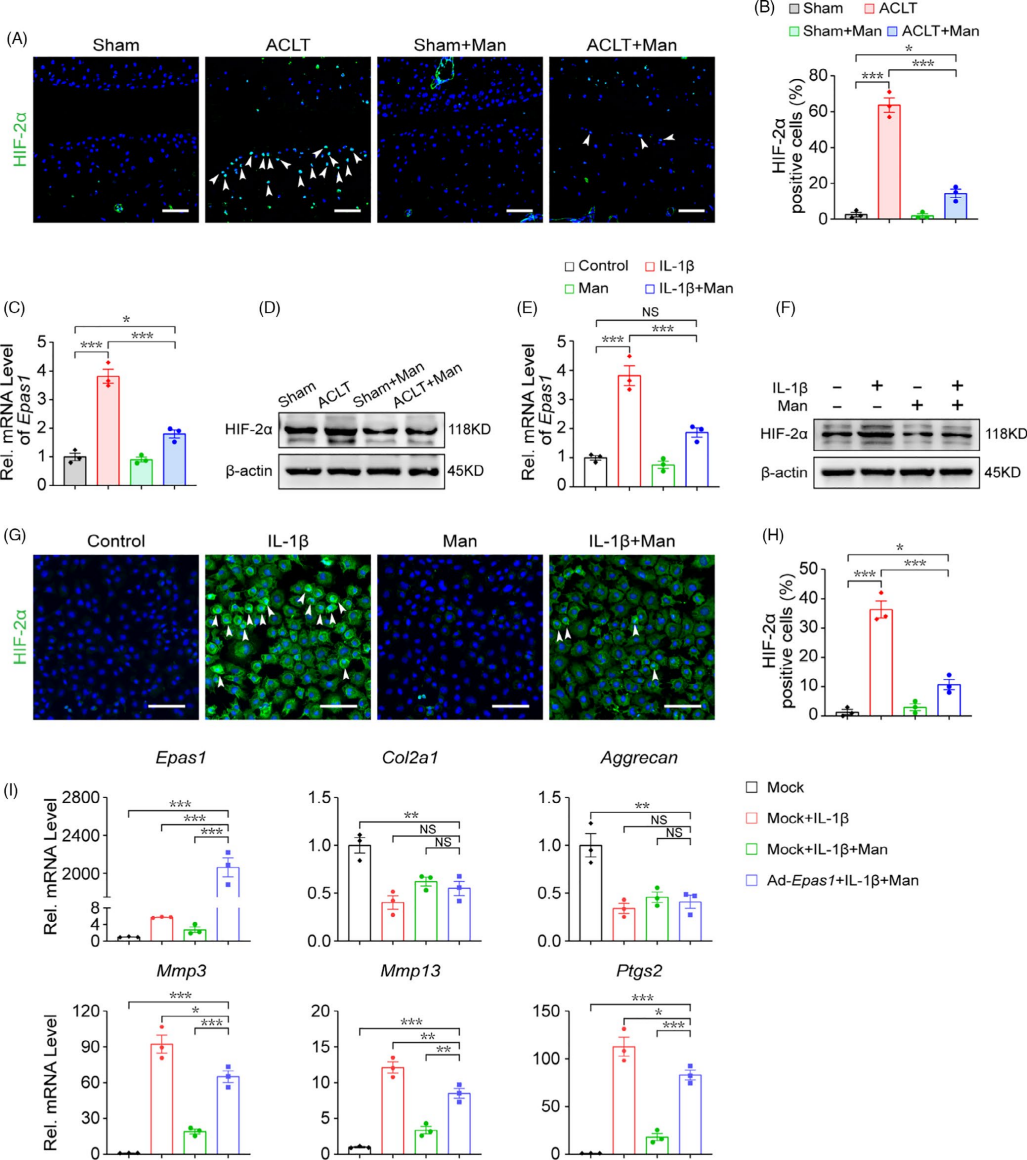
D‐mannose alleviates cartilage degeneration by suppressing HIF‐2α. (A and B) (A) Representative immunofluorescence staining of HIF‐2α in knee joint 4 weeks post‐surgery and (B) quantification. Arrow heads indicated positive cells. *n* = 3. Scale bars, 100 μm. (C and D) (C) Quantitative RT‐PCR (D) Western blot analyses of Epas1 expression of knee joint cartilage tissue. *n* = 3. (E and F) (E) Quantitative RT‐PCR and (F) Western blot analyses of the Epas1 expression of chondrocytes 24 h post indicated treatments. *n* = 3. (G and H) (G) Representative immunofluorescence staining of HIF‐2α of chondrocytes 24 h post indicated treatments and (H) quantification. *n* = 3. Scale bars, 100 μm. (I) Quantitative RT‐PCR analyses of the anabolic and catabolic gene expression of chondrocytes 24 h post indicated treatments. *n* = 3. Man, D‐mannose. All quantified data are shown as mean ± SEM; NS, not significant, *p < 0.05, **p < 0.01, ***p < 0.001 by one‐way ANOVA followed by the Tukey‐Kramer test

HIF‐2α is a key catabolic mediator regulated by NF‐κB signalling in OA progression.^23^, ^24^, ^35^ As is revealed by histological assay, ACLT strongly induced p65 phosphorylation while in contrast, phosphorylation of p65 in cartilages from D‐mannose‐treated ACLT group was significantly reduced (Figure S5A,B). Consistently, in cultured chondrocytes, p65 nuclear translocation ratio was dramatically upregulated by IL‐1β treatment so did the expression level, which were suppressed by D‐mannose treatment (Figure S5C–E). We next investigated the change of HIF‐2α expression in response to D‐mannose in cartilage degeneration models in vivo and in vitro. In vivo, compared with the sham group, the percentage of HIF‐2α‐positive chondrocytes in the ACLT group increased dozens of times, but reverted to a level comparable with that of the sham group when D‐mannose was applied (Figure 4A,B). Furthermore, quantitative RT‐PCR and Western blot analysis revealed a marked decrease of HIF‐2α in the ACLT + Man group versus the untreated ACLT group (Figure 4C, D). Consistently, the HIF‐2α level in cultured chondrocytes was elevated by IL‐1β treatment whereas D‐mannose treatment had the opposite effect (Figure 4E–H).

To further assess the role of HIF‐2α in the inhibition of cartilage degeneration by D‐mannose in vitro, we employed an *Epas1*‐encoding small interfering RNA (si*Epas1*) to knock down HIF‐2α and conversely an adenovirus (Ad‐*Epas1*) to overexpress it. As expected, si*Epas1* drastically blocked the elevated expression of catabolic genes (*Mmp3*, *Mmp13 and Ptgs2*) in chondrocytes induced by IL‐1β (Figure S6A,B), while Ad‐*Epas1* resulted in enhanced expression of *Mmp3*, *Mmp13* and *Ptgs2* in chondrocytes (Figures S6C,D). These confirmed the previously reported role of HIF‐2α in chondrocyte degeneration.^24^, ^36^ Notably, quantitative RT‐PCR analysis revealed that HIF‐2α overexpression abolished D‐mannose‐mediated suppression of *Mmp3*, *Mmp13* and *Ptgs2* (Figure 4I). Together, these data suggested that D‐mannose alleviates cartilage degeneration by suppressing HIF‐2α via NF‐κB signalling.

### 3.5 D‐mannose decreases chondrocyte ferroptosis sensitivity via inhibiting HIF‐2α expression

Since D‐mannose impedes HIF‐2α activation and cartilage degeneration, we tested whether HIF‐2α is involved in D‐mannose‐mediated chondrocyte ferroptosis inhibition. First, we silenced HIF‐2α in chondrocytes via siRNA transfection and found that HIF‐2α inhibition suppressed IL‐1β‐elevated lipid peroxidation significantly while upregulating *Gpx4* and *Scl7a11* expression (Figures S7A,B). Furthermore, si*Epas1* promoted *Cpt1a* and decreased *Hilpda* expression, indicating suppressed ferroptosis sensitization (Figure S7C).^22^ In accordance, overexpression of HIF‐2α with Ad‐*Epas1* compromised the viability of chondrocytes; ferroptosis suppressor genes *Gpx4* and *Scl7a11*, and β‐oxidation enzyme gene *Cpt1a*; and augmented the levels of lipid peroxidation by‐product MDA and ferroptosis sensitivity gene *Hilpda* (Figure S7D–G). Besides, overexpression of HIF‐2α significantly increased the expression of *Nox2* but neither the expression nor activity of SOD changed, indicating a final outcome of enhanced oxidative stress (Figure S7H, I). It is known that HIF‐2α affects the susceptibility of cancer cells to ferroptosis.^21^, ^22^ Our results suggested that HIF‐2α also plays a role in potentiating the susceptibility of chondrocytes to ferroptosis.

**FIGURE 5:**
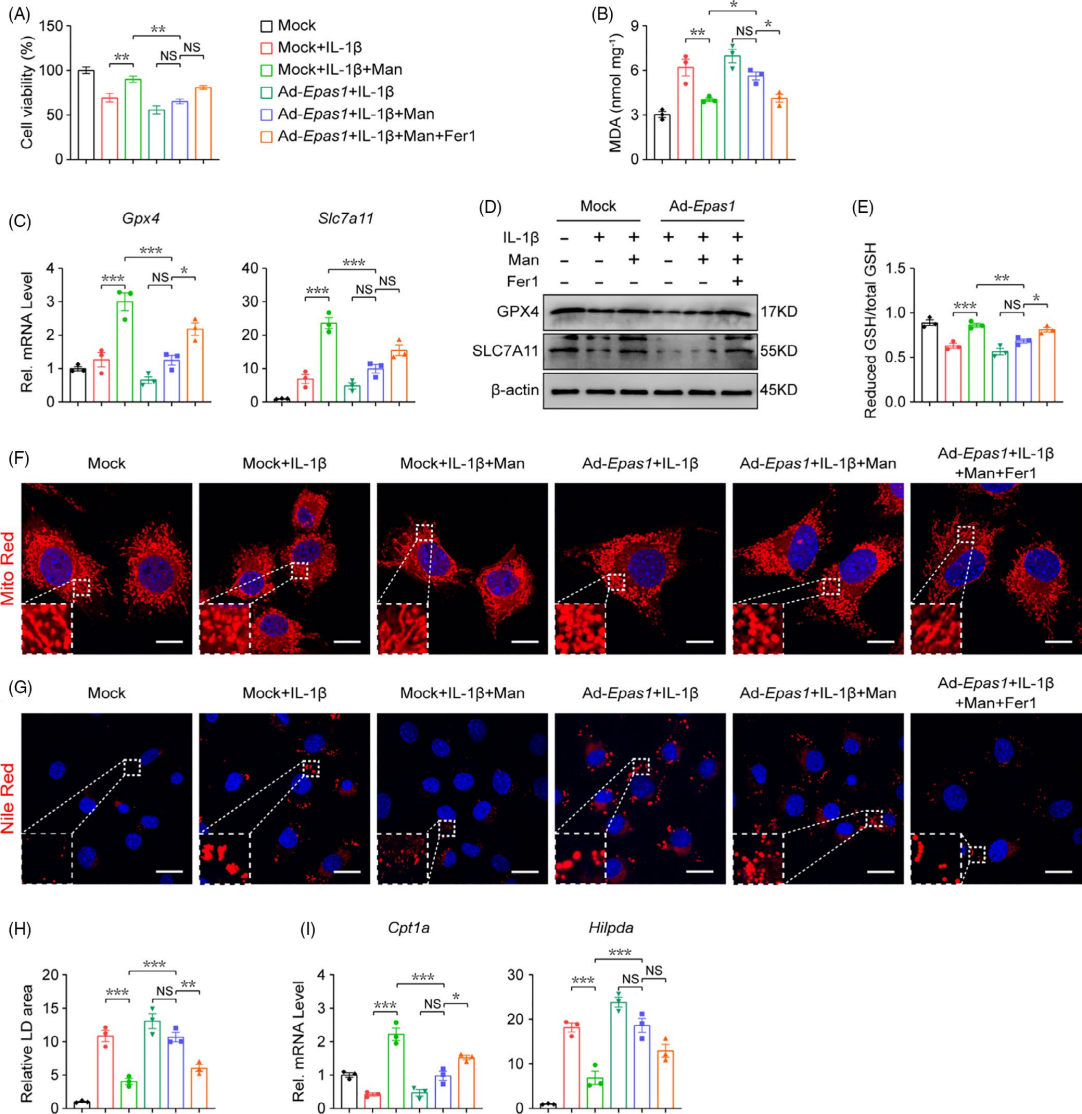
D‐mannose decreases chondrocyte ferroptosis sensitivity via inhibiting HIF‐2α expression. (A) Chondrocytes cell viability determined by CCK‐8 assay 48 h post indicated treatment. *n* = 4. (B) MDA measurement 24 h post indicated treatment. *n* = 3. (C and D) (C) Quantitative RT‐PCR and (D) Western blotting analyses of the Gpx4 and Slc7a11 of chondrocytes 24 h post indicated treatments. *n* = 3. (E) Measurement of GSH content 24 h post indicated treatment. *n* = 3. (F) Representative images of chondrocytes 24 h post indicated treatments visualized with MitoTracker Red staining. Dotted areas were magnified in the left bottom. Scale bars, 20 μm. (G and H) (G) Representative images of chondrocytes 24 h post indicated treatments stained with Nile Red to detect LDs and (H) quantification. Dotted areas were magnified in the left bottom. *n* = 3. Scale bars, 40 μm. (I) Quantitative RT‐PCR analyses of the *Cpt1a* and *Hilpda* gene expression of chondrocytes 24 h post indicated treatments. *n* = 3. Man, D‐mannose. All quantified data are shown as mean ± SEM; NS, not significant, *p < 0.05, **p < 0.01, ***p < 0.001 by one‐way ANOVA followed by the Tukey‐Kramer test

Second, we assessed whether HIF‐2α plays a vital role in D‐mannose‐induced chondrocyte ferroptosis resistance. Notably, HIF‐2α overexpression in chondrocytes reversed the protective effect of D‐mannose on IL‐1β‐impaired chondrocyte survival (Figure 5A). Meanwhile, in Ad‐*Epas1*‐treated chondrocytes, D‐mannose failed to decrease level of MDA enhanced by IL‐1β (Figure 5B). Also, the D‐mannose‐induced higher expression of Gpx4 and Scl7a11 at both the RNA and protein levels was eliminated by Ad‐*Epas1* (Figure 5C,D). In line with that, HIF‐2α overexpression caused a reduction in the ratio of reduced GSH out of total GSH (Figure 5E). D‐mannose failed to improve the mitochondrial impairment of IL‐1β‐treated HIF‐2α‐overexpressing chondrocytes (Figure 5F) and had no significant effect on lipid degeneration in HIF‐2α‐overexpressing chondrocytes as it altered neither LD deposition nor the expression of *Cpt1a* or *Hilpda* (Figure 5G–I). Moreover, the ferroptosis inhibitor ferrostatin‐1 (Fer‐1) partly offset the effect of HIF‐2α overexpression on D‐mannose‐induced ferroptosis resistance (Figure 5A–I). Collectively, these data suggested that D‐mannose inhibits ferroptosis in a HIF‐2α‐dependent manner.

### 3.6 **D‐mannose‐induced downregulation of HIF‐2**α **inhibits OA progression through suppressing chondrocyte ferroptosis**

**FIGURE 6:**
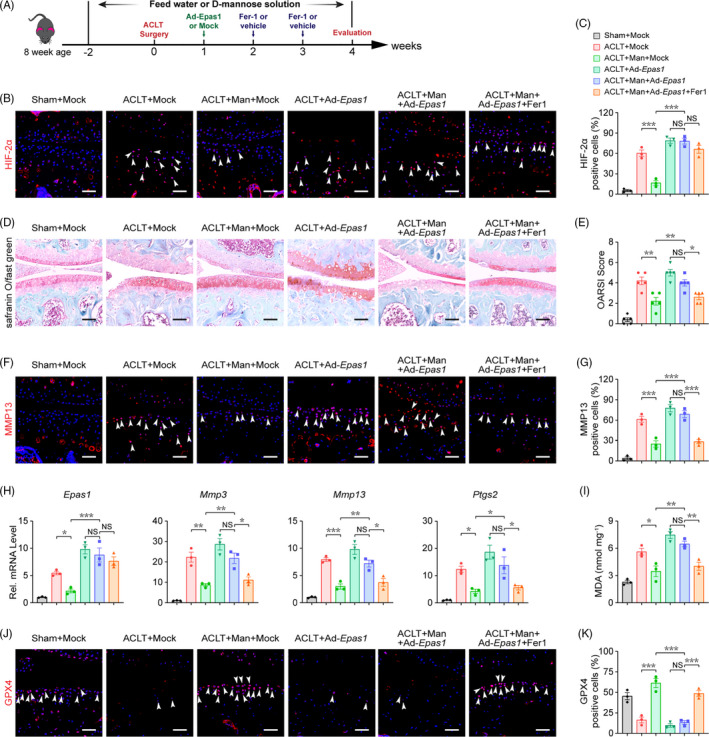
D‐mannose‐induced downregulation of HIF‐2α inhibits OA progression through suppressing chondrocyte ferroptosis. (A) Schematic model of the time course for establishment of the anterior cruciate ligament transection (ACLT) model of OA mouse with/without D‐mannose administration and adenovirus/ferrostain‐1 (Fer‐1) injection. (B and C) (B) Representative immunofluorescence staining of HIF‐2α in knee joint 4 weeks post‐surgery and (C) quantification. Arrow heads indicated positive cells. *n* = 3. Scale bars, 100 μm. (D) Representative safranin O/fast green staining of sham and ACLT‐induced OA mice treated with/without D‐mannose administration and adenovirus/Fer‐1 injection 4 weeks post‐surgery. Scale bars, 200 μm. (E) Osteoarthritis Research Society International (OARSI) score evaluated based on Safranin O/fast green staining 4 weeks post‐surgery. *n* = 5. (F and G) (F) Representative immunofluorescence staining of MMP13 in knee joint 4 weeks post‐surgery and (G) quantification. Arrow heads indicated positive cells. *n* = 3. Scale bars, 100 μm. (H) Quantitative RT‐PCR analyses of the catabolic gene expression of knee joint cartilage tissue 4 weeks post‐surgery. *n* = 3. (I) MDA measurement of knee joint cartilage tissue. *n* = 3. (J) Representative immunofluorescence staining of GPX4 in knee joint 4 weeks post‐surgery and (K) quantification. Arrow heads indicated positive cells. *n* = 3. Scale bars, 100 μm. Man, D‐mannose. All quantified data are shown as mean ± SEM; NS, not significant, *p < 0.05, **p < 0.01, ***p < 0.001 by one‐way ANOVA followed by the Tukey‐Kramer test

**FIGURE 7:**
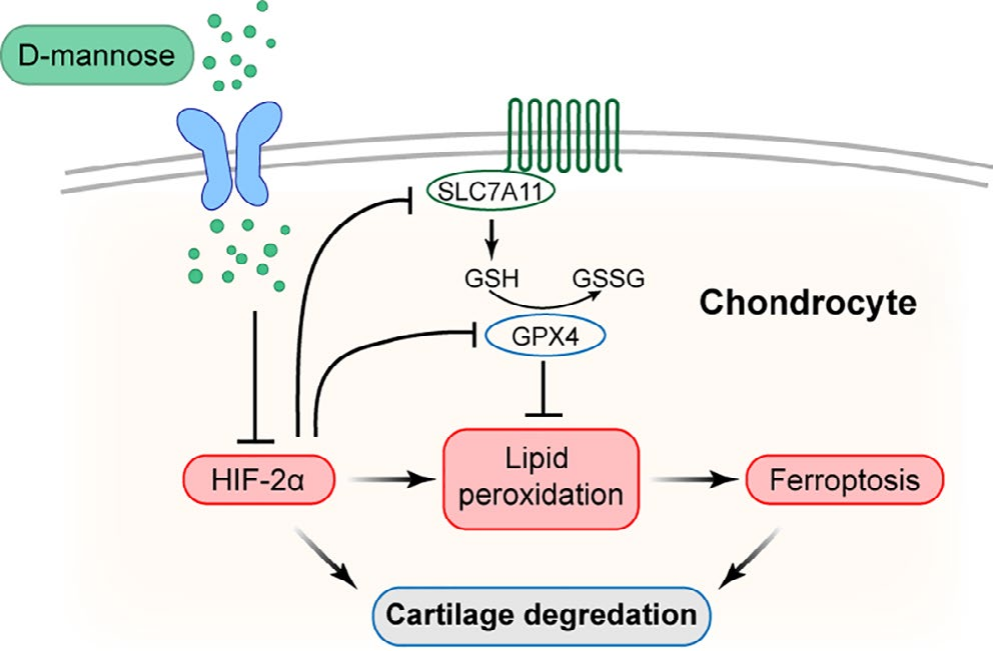
Model of D‐mannose ameliorates osteoarthritis progression by inhibiting chondrocyte ferroptosis in a HIF‐2α‐dependent manner. D‐mannose inhibited chondrocyte ferroptosis by suppressing HIF‐2α during OA progression, leading to the prevention of cartilage degeneration

Our findings in vitro prompted us to further analyse the function of HIF‐2α downregulation in D‐mannose‐induced chondrocyte resistance to ferroptosis and OA progression in vivo. Consistent with previous studies which indicated the role of chondrocyte ferroptosis in OA progression,^9^, ^10^ immunofluorescence staining described above revealed a striking reduction of the key ferroptosis inhibitor, GPX4, in the articular chondrocytes upon ACLT surgery (Figure 3A). To examine whether D‐mannose‐induced inhibition of HIF‐2α would protect osteoarthritic cartilage by suppressing chondrocyte ferroptosis, we fed mice with D‐mannose solution or water as previously described and then performed ACLT surgery. Adenovirus with (Ad‐*Epas1*) or without (Mock) *Epas1* was injected intraarticularly (Figure 6A). In ACLT mice with or without Ad‐*Epas1* injection, the HIF‐2α expression in osteoarthritic chondrocytes was both significantly upregulated to almost level compared with control. However, Ad‐*Epas1* injection significantly offset D‐mannose‐induced HIF‐2α inhibition in ACLT mice 4 weeks post‐surgery (Figure 6B, C). Consistent with our findings in vitro (Figure 4I), when comparing the ACLT+Man+Ad‐*Epas1* group with the ACLT+Man group, we observed that the overexpression of HIF‐2α by Ad‐*Epas1* injection abolished the protective effect of D‐mannose on OA progression and cartilage degeneration in vivo, as indicated by safranin O staining and OARSI score (Figure 6D, E). These observations were further confirmed by MMP13 staining and qPCR analysis of *Mmp3*, *Mmp13* and *Ptsg2* (Figure 6F–H). Furthermore, in the ACLT+Man+Ad‐*Epas1* group, the ferroptosis activity was significantly re‐enhanced compared with the ACLT + Man group, as indicated by upregulation of MDA and reduced expression of GPX4 (Figure 6I,K). However, based on all the indicators above, in ACLT mice with Ad‐*Epas1* injection, D‐mannose no more extended any effects on chondrocytes and cartilage (Figure 6B–K). In addition, intra‐articular injection of Fer‐1 suppressed the effects of Ad‐*Epas1* on ferroptosis activity and prevented cartilage destruction (Figure 6B‐K). Together, these in vivo data strongly suggested that D‐mannose exerts a chondroprotective effect and alleviates OA progression by suppressing HIF‐2α‐mediated chondrocyte sensitivity to ferroptosis (Figure 7).

## 4 DISCUSSION

OA is the most prevalent joint disease and remains on the rise; however, there is still no treatment that can block OA progression. In this study, we demonstrated that D‐mannose, a natural C‐2 epimer of glucose, alleviated OA development in vivo and exerted a chondroprotective effect in vitro by inhibiting the sensitivity of chondrocytes to ferroptosis. HIF‐2α played a central role in this process. Upregulation of HIF‐2α induced chondrocyte ferroptosis and blocked the chondroprotective effects of D‐mannose.

Previous research has shown that D‐mannose has anti‐inflammatory effects and is therapeutically effective as a non‐antibiotic treatment for recurrent urinary tract infections. As for OA, by employing a monosodium iodoacetate‐treated rat model, Lin et al.^16^ demonstrated that gavage of D‐mannose can suppress OA progression by upregulating anabolism and downregulating catabolism of chondrocytes. Likewise, in this study, D‐mannose succeeded in downregulating pro‐inflammatory factors both in vivo and in vitro, and we found a similar phenotypic effect of D‐mannose in our surgically induced OA model. But here, D‐mannose preserved COL2A1 expression and downregulated MMP13 levels in vivo mainly by inhibiting chondrocyte catabolism, which somewhat differed from prior findings. Moreover, in Lin's study, only a moderate dose (1.25 g/kg/day) of D‐mannose suppressed OA development. Considering the methodological differences, these studies together indicate that the pathogenic mechanism of OA, cell sensitivity, mode, dose and duration of administration could alter the biological effect of D‐mannose and should be taken into further consideration to achieve successful management of OA.

It is known that articular cartilage damage is the most obvious pathologic feature leading to joint dysfunction. Earlier research reported several ferroptosis‐related features, such as abnormal iron metabolism,^10^, ^11^, ^37^, ^38^ lipid peroxidation^39^, ^40^ and mitochondrial dysfunction,^41^, ^42^ are closely correlated with accelerating cartilage destruction. It is only recently that Yao et al.^9^ first demonstrated that chondrocyte ferroptosis contributes to the progression of osteoarthritis. In fact, besides ferroptosis, other forms of cell death, including necrosis, apoptosis and autophagy, of chondrocytes all interplay with cartilage degradation and OA progression.^8^, ^43^, ^44^ Our data suggested that D‐mannose functions as an effective ferroptosis suppresser of chondrocyte mediated by HIF‐2α which contributes to protect osteoarthritic cartilage from destruction. Notably, in another study focussing on D‐Mannose functions on OA, Lin et al.^16^ found that D‐Mannose enhanced chondrocyte autophagy via the AMPK pathway indicating a potential relationship between mechanisms of D‐mannose therapeutic effect in OA. In fact, increasing body of evidence reflects that there exists an intrinsic connection between autophagy and ferroptosis. On the one hand, some studies identified autophagy is as an upstream mechanism in the induction of ferroptosis by regulating cellular iron homeostasis and cellular ROS generation.^45^, ^46^ On the other hand, ferroptosis regulators are also involved in the control of autophagy dependent on the context.^47^ Besides, recent studies revealed that HIF‐2α and AMPK pathway participated in these two forms of cell death respectively. For example, Lee et al.^48^ found that AMPK activation could inhibit ferroptosis under energy stress by mediating phosphorylation of acetyl‐CoA carboxylase (ACC) and polyunsaturated fatty acid biosynthesis. By activating Akt‐1 and mTOR activities, HIF‐2α robustly downregulates autophagy in maturing chondrocytes.^49^ However, the relationship between HIF‐2α and AMPK pathway is still unclear and whether they are overlapped or functions independently and whether there exist other mechanisms controlling chondrocyte fate that contribute to the D‐mannose therapeutic effect in OA need more experimental evidence in the future.

It is well acknowledged that the activity of HIF‐2α, an extensive regulator of OA, and its upstream regulator, NF‐κB, increases during OA development.^20^, ^23^, ^24^ So far, several mechanisms by which HIF‐2α regulates OA have been demonstrated. On one hand, HIF‐2α directly induces higher expression of catabolic factors, leading to articular cartilage destruction.^24^ On the other hand, HIF‐2α levels in human and mouse OA chondrocytes are highly associated with kinds of regulated cell death of chondrocytes.^50^ Meanwhile, HIF‐2α potentiates Fas‐mediated chondrocyte apoptosis and decreases expression of autophagy during OA development.^49^, ^51^ Here, besides findings consistent with previous ones establishing that HIF‐2α is a catabolic regulator of osteoarthritic cartilage destruction, we first reported a novel mechanism of HIF‐2α during OA progression by which it potentiates cell death via lipid oxidation, ROS accumulation and ferroptosis regulators. Our work not only provides a novel finding that HIF‐2α mediates osteoarthritic cartilage degeneration via creating a ferroptosis‐susceptible cell state of chondrocytes, which broadens the understanding of the potential therapeutic role for HIF‐2α in OA management, but also brings insights into the mechanisms underlying ferroptosis susceptibility within an OA microenvironment.

Notably, previous studies highlighted the involvement of D‐mannose in immunomodulation at the cellular and molecular levels and confirmed that D‐mannose can suppress the immunopathology of autoimmune diabetes, airway inflammation and lupus.^13^, ^52^ In macrophages, D‐mannose interferes with glucose metabolism by raising intracellular mannose‐6‐phosphate levels to inhibit macrophage activation and IL‐1β production.^14^, ^17^ Zhang et al.^13^, ^18^ reported that by upregulation of reactive oxygen species generated by increased fatty acid oxidation, D‐mannose promotes TGF‐β activation to stimulates Treg cell differentiation. It cannot be neglected that an activation of innate and adaptive immune responses contribute to the initiation and sustaining of OA, during which immune cells infiltrate synovium and act as a main contributor for the release of disease‐specific cytokines and chemokines.^53^, ^54^ Typically, macrophages can stimulate the release of pro‐inflammatory cytokines like IL‐1β and TNF‐α and other catabolic and anabolic mediators involved OA pathology. Besides, T cells are responsible for increased infiltration of macrophages by inducing macrophage inflammatory protein‐1γ and can interact with chondrocytes through cell surface molecules to upregulate MMPs release.^55^ Considering the tight connection of D‐mannose, immune and OA progression, we consider that beyond the aim of the present work, future investigations are foreseen to determine whether, and the extent to which, immunomodulation induced by mannose administration in mice might contribute to OA amelioration.

Collectively, our investigations have revealed the previously unrecognized role of D‐mannose in attenuating OA progression via inhibiting HIF‐2α‐induced ferroptosis, indicating a novel, safe and effective therapeutic strategy for OA and many other ferroptosis‐related diseases.

## CONFLICT OF INTEREST

The authors declare no competing interests.

## AUTHOR CONTRIBUTIONS

J.L. and J.W. conceived and designed the project. X.Z. and Y.Z. performed the experiments; W.S., Z.Z., J.L., W.Y., X.Y. and W.S. contributed to data acquisition. X.Z., Y.Z., Z.Z. and W.S. analysed the data; X.Z. and Y.Z. wrote and edited the manuscript; Y.Y., J.L. and J.W. contributed to the manuscript revision. J.L. and J.W. supervised the research. All authors read and approved the final paper.

## Supporting information

Supplementary Material

## Data Availability

The data that support the findings of this study are available from the corresponding author upon reasonable request.
