# Supplementary material for: D‐mannose alleviates osteoarthritis progression by inhibiting chondrocyte ferroptosis in a HIF‐2α‐dependent manner
Source: Cell Prolif. 2021 Sep 25;54(11):e13134. doi: 10.1111/cpr.13134 (PMC8560605; doi:10.1111/cpr.13134)
Supplement: Supplementary file 1 — Supplementary Material [file CPR-54-e13134-s001.docx]

Supplementary information for:

D-Mannose Alleviates Osteoarthritis Progression by Inhibiting Chondrocyte Ferroptosis in a HIF-2α-Dependent Manner

**TABLE OF CONTENTS**

**SUPPLEMENTARY FIGURES……………………………………………………… 2**

**SUPPLEMENTARY TABLES……………………………………………………….. 10**

# SUPPLEMENTARY FIGURES


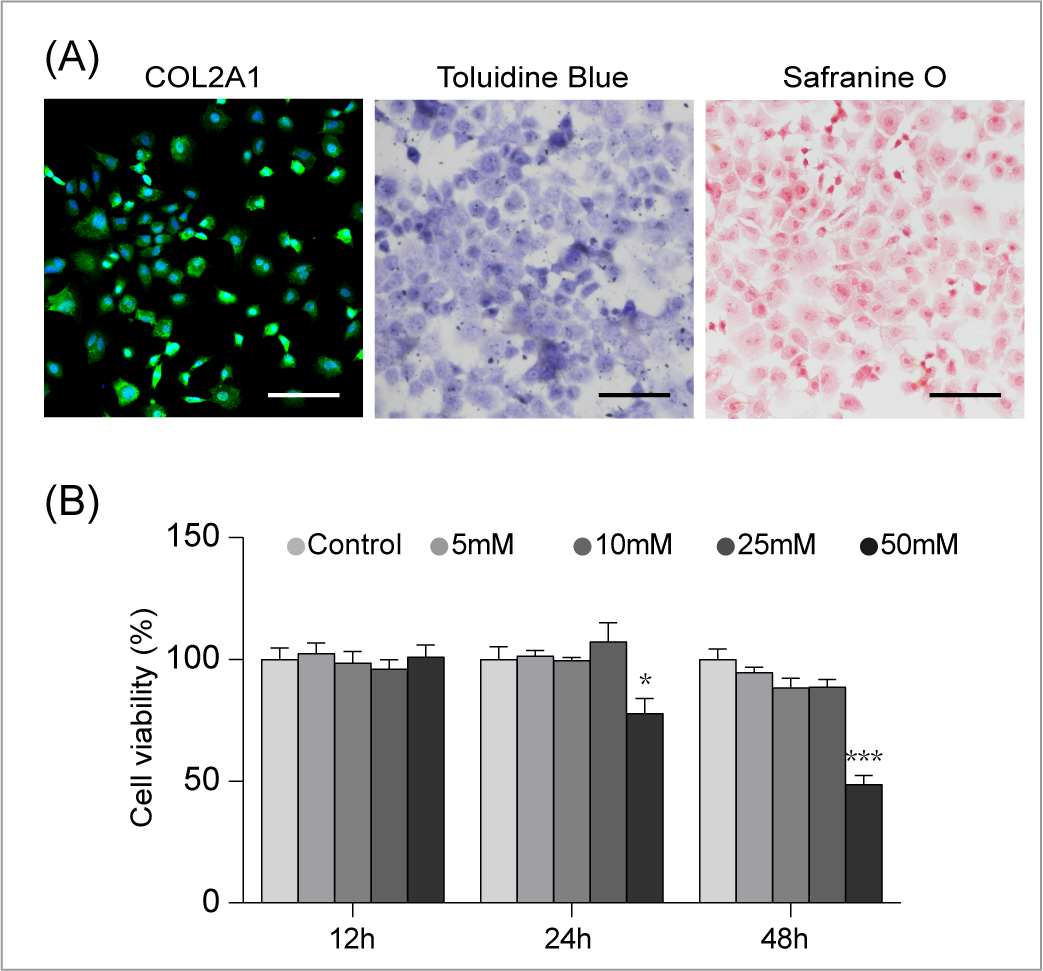


**Figure S1. Moderate D-mannose treatment is safe to chondrocytes.**

(A) Verification of mouse primary chondrocytes with COL2A1, toluidine blue, and safranin O staining. Scale bars, 100 μm. (B) Chondrocyte’s viability determined by CCK-8 assay. Chondrocytes were incubated with various D-mannose (Man) concentrations (0, 5, 10, 25, and 50 mM) and various time periods (12, 24, and 48 h). *n* = 4. All quantified data are shown as mean ± SEM; NS, not significant, **P* < 0.05, ***P* < 0.01, ****P* ＜ 0.001 by one-way ANOVA followed by the Tukey- Kramer test.

**
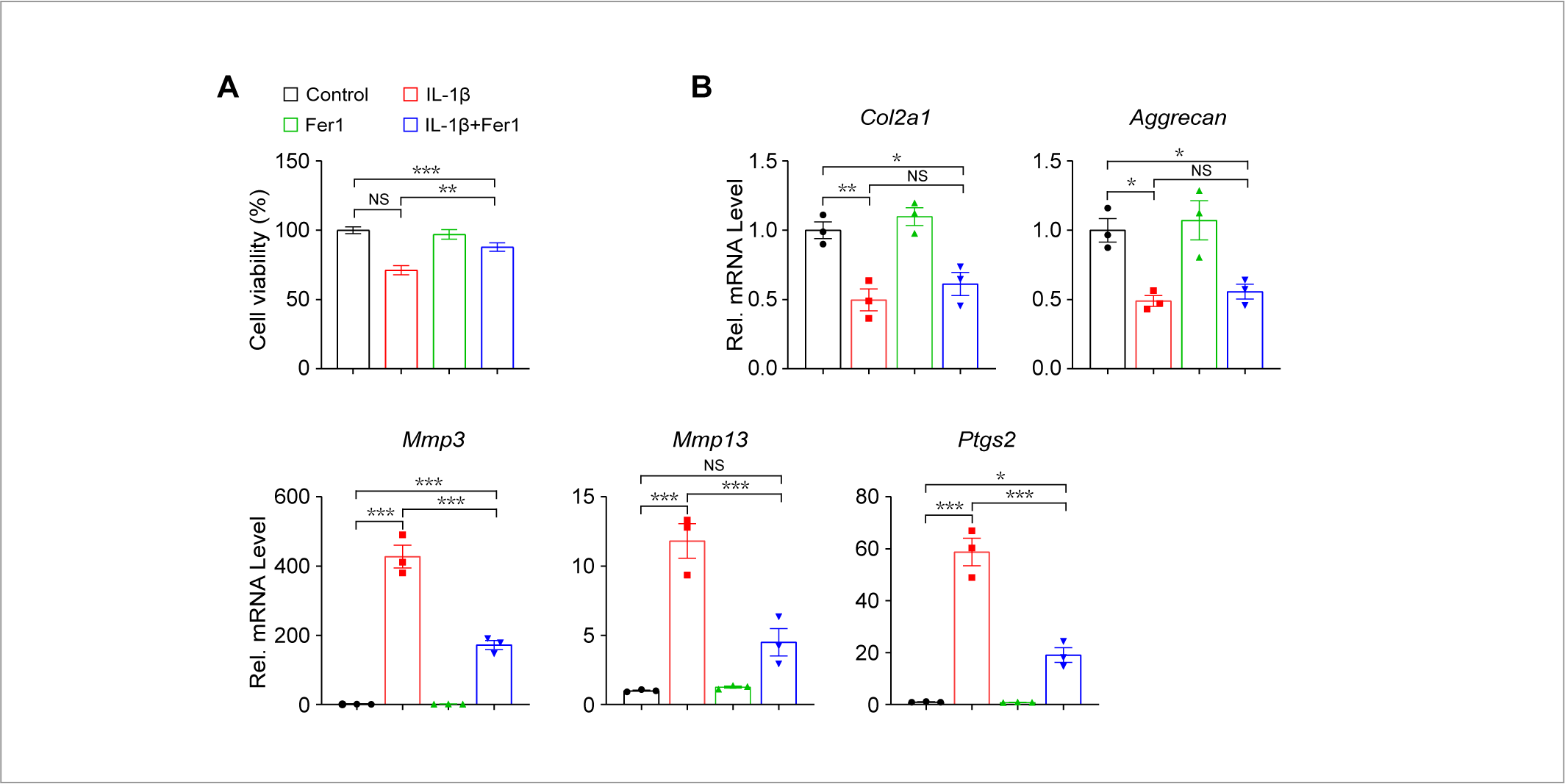
**

**Figure S2. Ferrostatin-1 attenuated the cytotoxicity and catabolic related gene expression induced by IL-1β in chondrocytes.**

(A) Chondrocyte’s viability determined by CCK-8 assay. *n* = 4. (B) Quantitative RT-PCR of *Col2a1, Aggrecan, Mmp3, Mmp13* and *Ptgs2* of chondrocytes 24 h post indicated treatments. *n* = 3. Fer1, Ferrostatin-1. All quantified data are shown as mean ± SEM; NS, not significant, **P* < 0.05, ***P* < 0.01, ****P* ＜ 0.001 by one-way ANOVA followed by the Tukey- Kramer test.

**
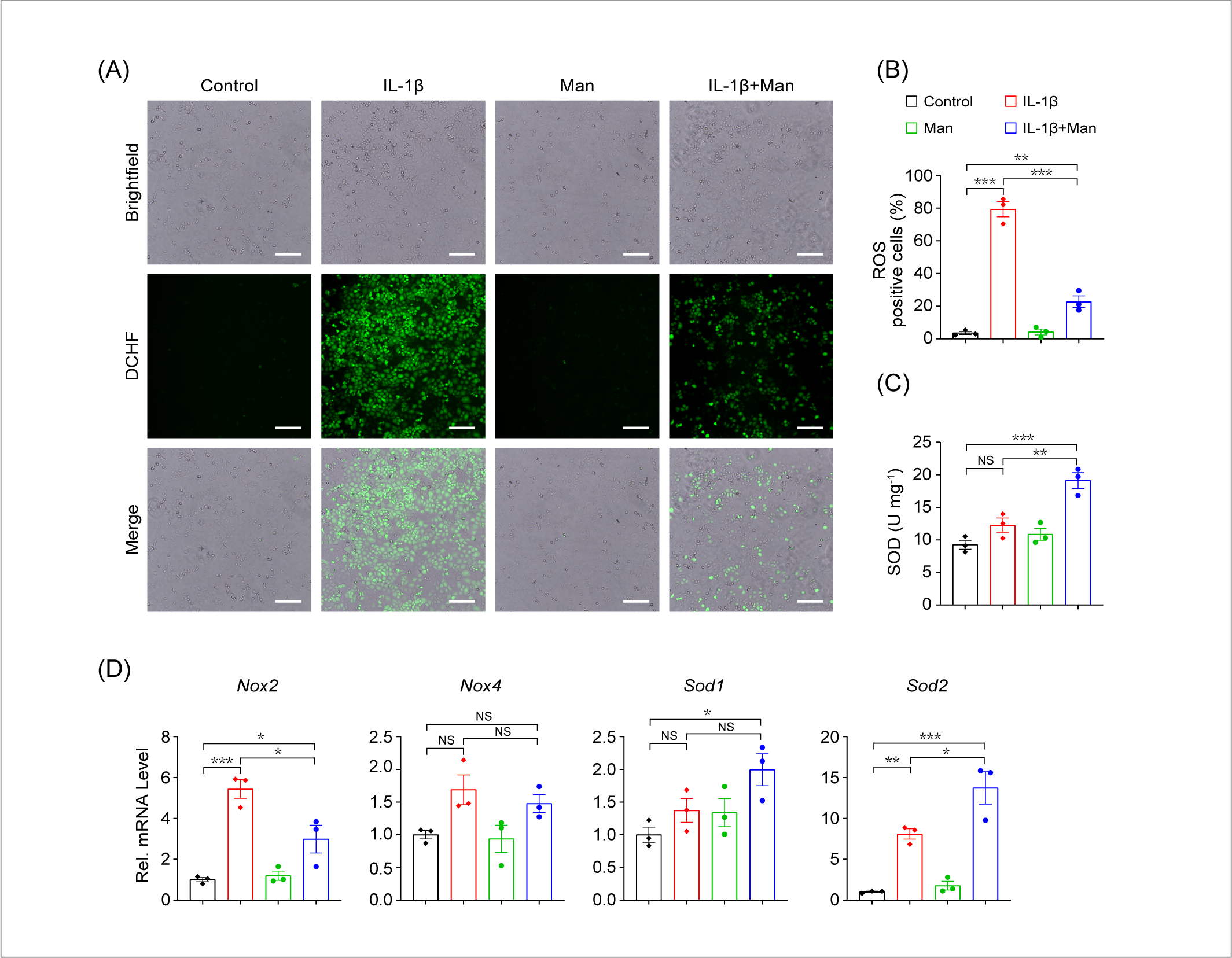
**

**Figure S3. D-mannose suppresses intracellular ROS of chondrocytes.**

(A and B) (A) DCFH-DA measurement and (B) quantitative analysis for ROS level of chondrocytes 24 h post indicated treatments. *n* = 3. Scale bars, 400 μm. (C) SOD activity measurement of chondrocytes 24 h post indicated treatments. *n* = 3. (D) Quantitative RT-PCR of *Nox2, Nox4, Sod1* and *Sod2* of chondrocytes 24 h post indicated treatments. *n* = 3. Man, D-mannose. Scale bars, 20 μm. All quantified data are shown as mean ± SEM; NS, not significant, **P* < 0.05, ***P* < 0.01, ****P* ＜ 0.001 by one-way ANOVA followed by the Tukey- Kramer test.

**
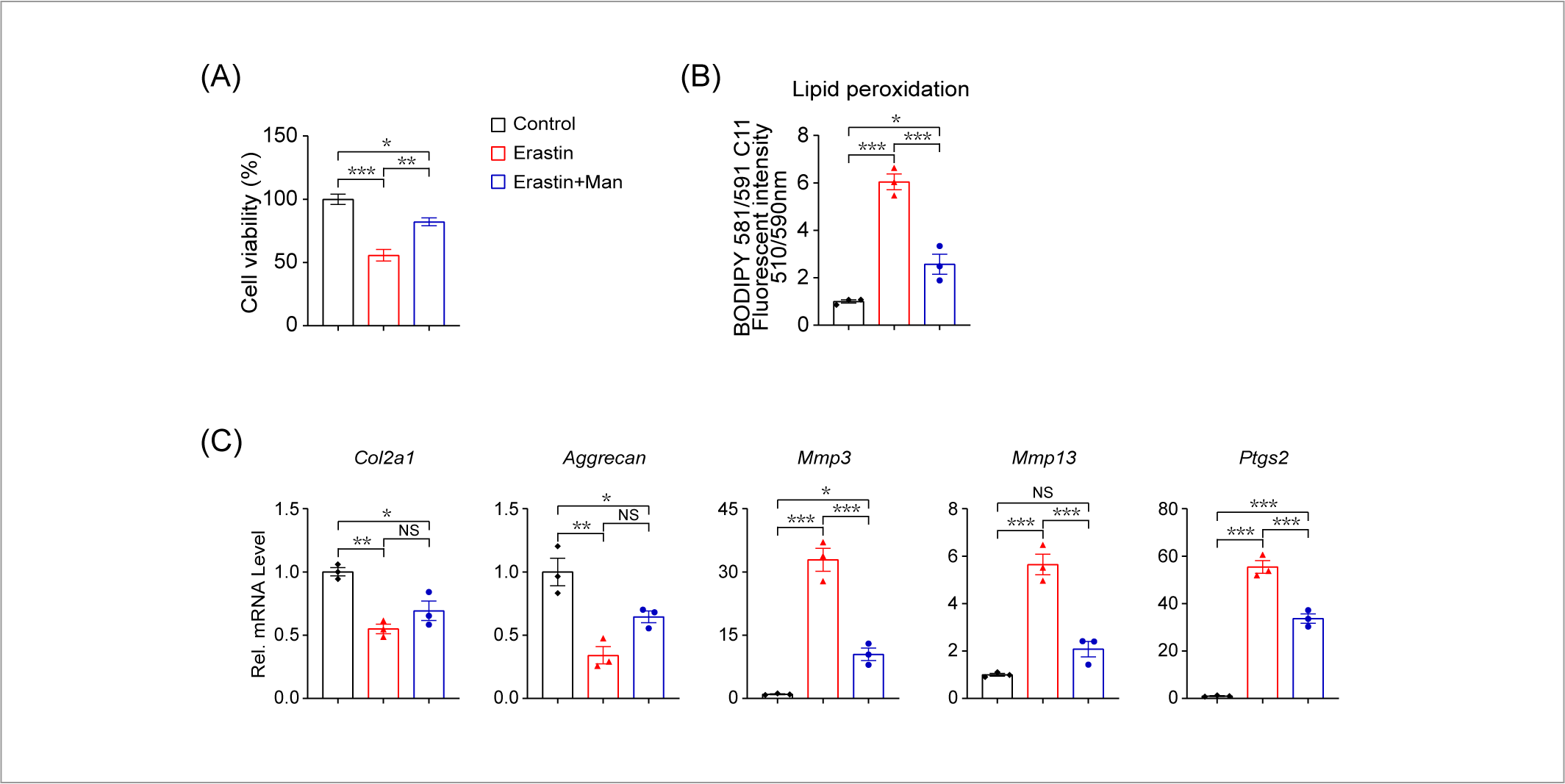
**

**Figure S4. D-mannose protects chondrocytes from ferroptosis and catabolism.**

(A) Cell viability determined by CCK-8 assay 48 h post post indicated treatments. *n* = 4. (B) Lipid peroxidation was determined using the BODIPY 581/591 C11 reagent in chondrocytes 24 h post indicated treatments. *n* = 3. (C) Quantitative RT-PCR analyses of the anabolic and catabolic gene expression of chondrocytes 24 h post indicated treatments. *n* = 3. All quantified data are shown as mean ± SEM; NS, not significant, **P* < 0.05, ***P* < 0.01, ****P* ＜ 0.001 by one-way ANOVA followed by the Tukey- Kramer test.

**
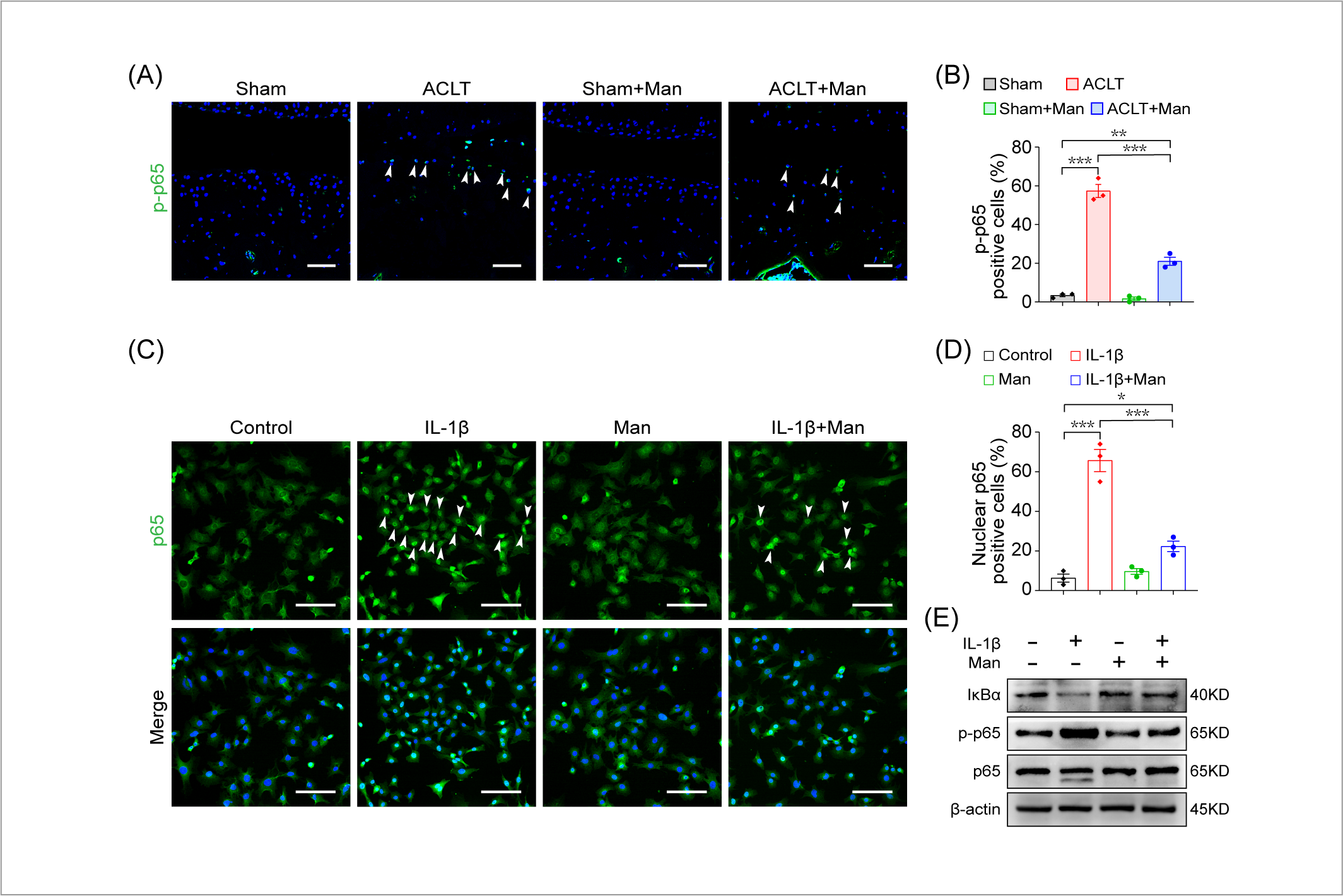
**

**Figure S5. D-mannose downregulates HIF-2α through NF-κB pathway.**

(A) Representative immunofluorescence staining of p-p65 in knee joint 4-weeks post-surgery and (B) quantification. Arrow heads indicated positive cells. *n* = 3. Scale bars, 100 μm. (C and D) (C) Representative immunofluorescence staining of p65 of chondrocytes 24 h post indicated treatments and (D) quantification. *n* = 3. Scale bars, 100 μm. (E) Western blot analyses of the level of IκBα, p-p65 and p65 of chondrocytes 24 h post indicated treatments. Man, D-mannose. All quantified data are shown as mean ± SEM; NS, not significant, **P* < 0.05, ***P* < 0.01, ****P* ＜ 0.001 by one-way ANOVA followed by the Tukey- Kramer test.

**
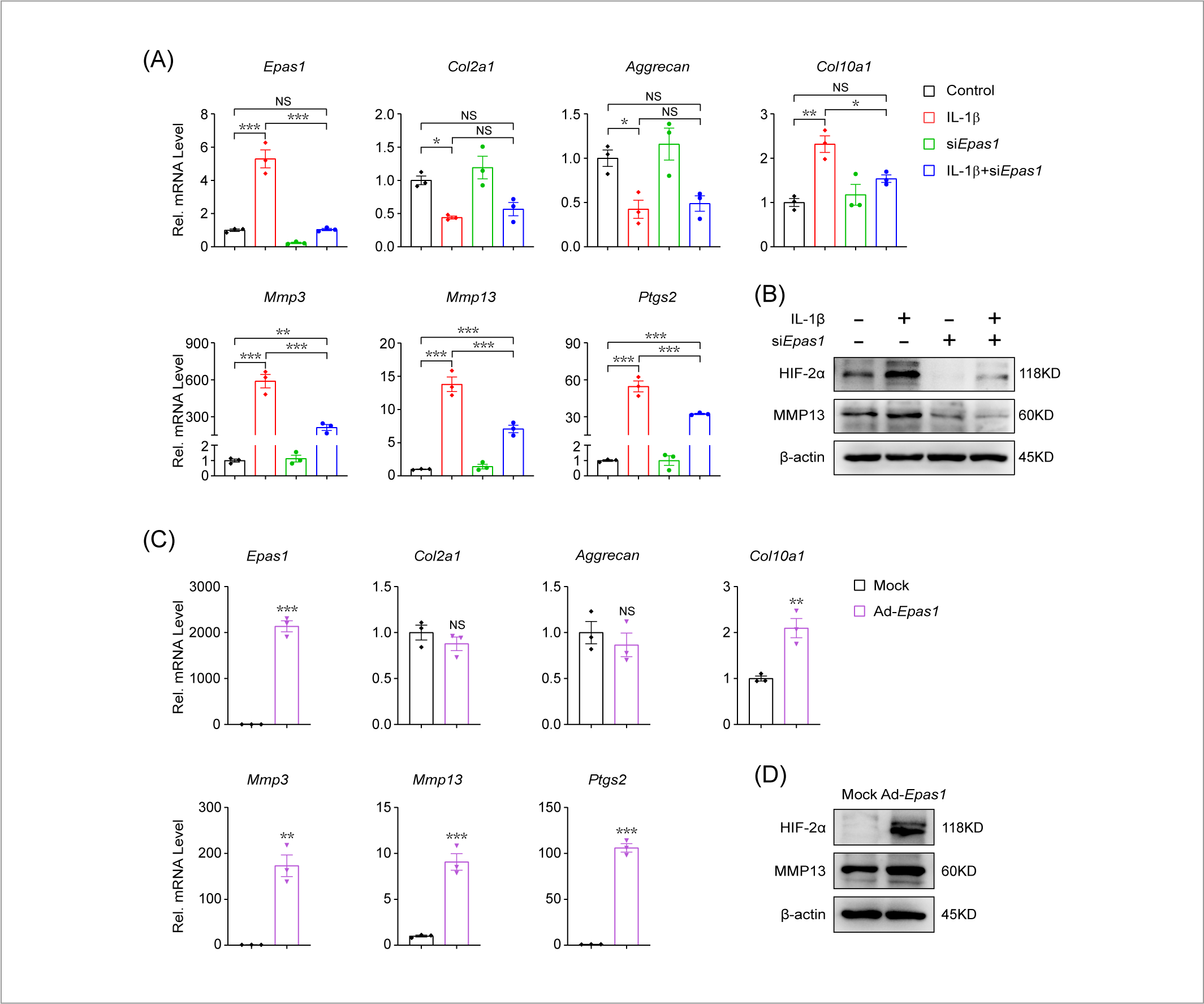
**

**Figure S6. HIF-2α is the key catabolic mediator of chondrocytes.**

(A and C) Quantitative RT-PCR of *Epas1* and the anabolic and catabolic gene expression of chondrocytes 24 h post indicated treatments. *n* = 3. (B and D) Western blotting analyses of HIF-2α and MMP13 expression of chondrocytes 24 h post indicated treatments. All quantified data are shown as mean ± SEM; NS, not significant, **P* < 0.05, ***P* < 0.01, ****P* ＜ 0.001 by (A)one-way ANOVA followed by the Tukey- Kramer test or (C) unpaired Student’s *t*-test.

**
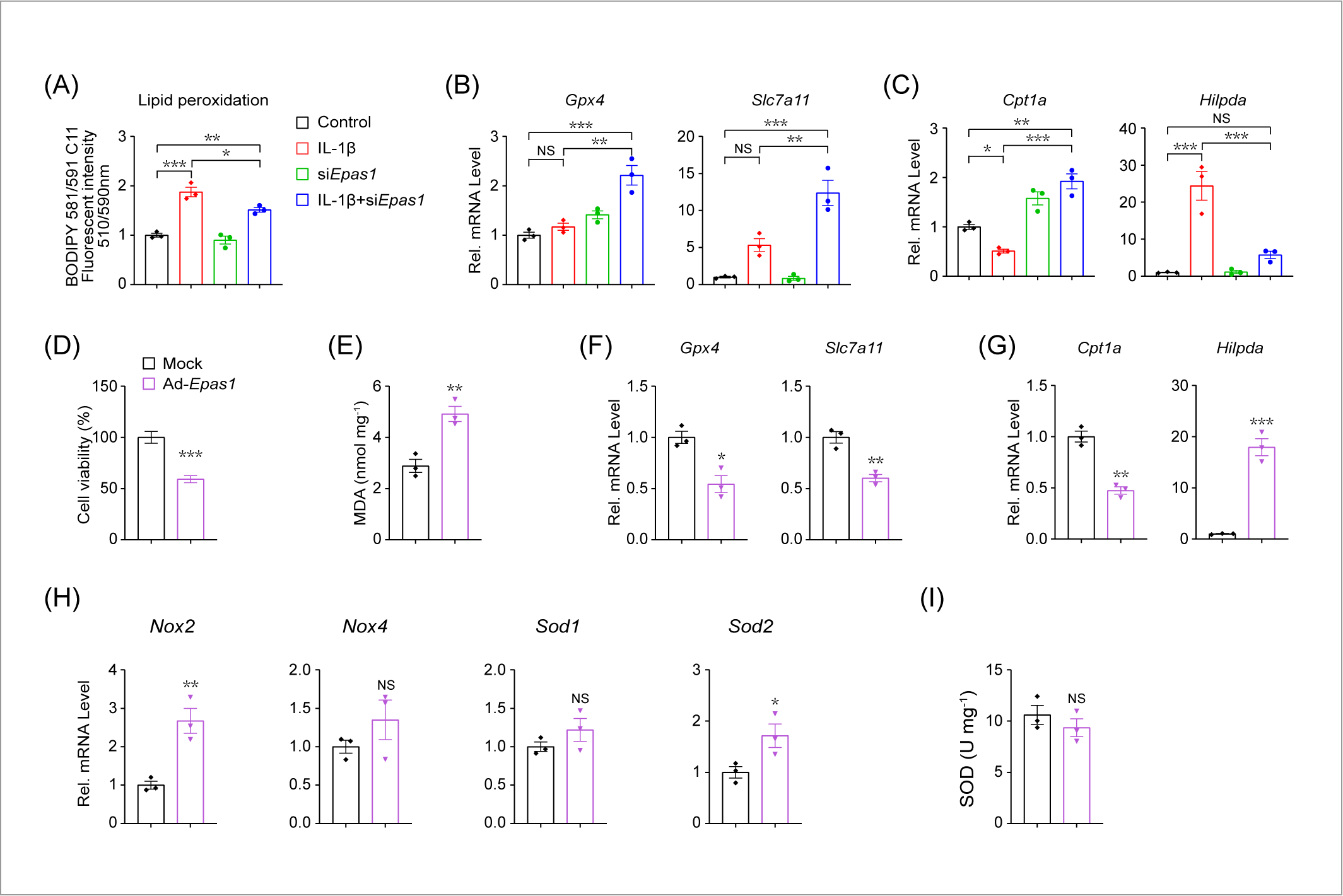
**

**Figure S7. HIF-2α potentiates chondrocyte ferroptosis.**

(A) Lipid peroxidation was determined using the BODIPY 581/591 C11 reagent in chondrocytes 24 h post indicated treatments. *n* = 3. (B) Quantitative RT-PCR analyses of the *Gpx4* and *Slc7a11* gene expression of chondrocytes 24 h post indicated treatments. *n* = 3. (C) Quantitative RT-PCR analyses of the *Cpt1a* and *Hilpda* gene expression of chondrocytes 24 h post indicated treatments. *n* = 3. (D) Cell viability determined by CCK-8 assay 48 h post infection. (E) MDA measurement of chondrocytes 24 h post infection. *n* = 3. (F) Quantitative RT-PCR analyses of the *Gpx4* and *Slc7a11* gene expression of chondrocytes 24 h post infection. *n* = 3. (G) Quantitative RT-PCR analyses of the *Cpt1a* and *Hilpda* gene expression of chondrocytes 24 h post infection. *n* = 3. (H) Quantitative RT-PCR analyses of the *Nox2, Nox4, Sod1* and *Sod2* of chondrocytes 24 h post infection. *n* = 3. (I) SOD activity measurement of chondrocytes 24 h post infection. *n* = 3. All quantified data are shown as mean ± SEM; NS, not significant, **P* < 0.05, ***P* < 0.01, ****P* ＜ 0.001 by (A-C) one-way ANOVA followed by the Tukey- Kramer test or (D-G) unpaired Student’s *t*-test.

# SUPPLEMENTARY TABLES

|  | Forward primers (5’-3’) | Reverse primers (5’-3’) |
| --- | --- | --- |
| Mouse *Epas1* | CUCAGUUACAGCCACAUCGUCACUG | CAGUGACGAUGUGGCUGUAACUGA |
| Control siRNA | CCUACGCCACCAAUUUCGU | ACGAAAUUGGUGGCGUAGG |

**Table S1. *Epas1* siRNA sequences.**

| Gene | Forward primers (5’-3’) | Reverse primers (5’-3’) |
| --- | --- | --- |
| *Col2a1* | GCAGAGATGGAGAACCTGGTA | AGCCTTCTCGTCATACCCT |
| *Aggrecan* | CCTGCTACTTCATCGACCCC | AGATGCTGTTGACTCGAACCT |
| *Col10a1* | GGGACTCACGTTTGGGTAGG | TCACATGGGAGCCACTAGGA |
| *Adamts5* | GGAGCGAGGCCATTTACAAC | CGTAGACAAGGTAGCCCACTTT |
| *Mmp3* | GGCCTGGAACAGTCTTGGC | TGTCCATCGTTCATCATCGTCA |
| *Mmp13* | CTTCTTCTTGTTGAGCTGGACTC | CTGTGGAGGTCACTGTAGACT |
| *Ptgs2* | CAGACAACATAAACTGCGCCTT | GATACACCTCTCCACCAATGACC |
| *Gpx4* | GATGGAGCCCATTCCTGAACC | CCCTGTACTTATCCAGGCAGA |
| *Slc7a11* | TGGGTGGAACTGCTCGTAAT | AGGATGTAGCGTCCAAATGC |
| *Cpt1a* | CTCCGCCTGAGCCATGAAG | CACCAGTGATGATGCCATTCT |
| *Epas1* | CTGAGGAAGGAGAAATCCCGT | TGTGTCCGAAGGAAGCTGATG |
| *Hilpda* | TGCTGGGCATCATGTTGACC | TGACCCCTCGTGATCCAGG |
| *Nox2* | TGTGGTTGGGGCTGAATGTC | CTGAGAAAGGAGAGCAGATTTCG |
| *Nox4* | GAAGGGGTTAAACACCTCTGC | ATGCTCTGCTTAAACACAATCCT |
| *Sod1* | AACCAGTTGTGTTGTCAGGAC | CCACCATGTTTCTTAGAGTGAGG |
| *Sod2* | CAGACCTGCCTTACGACTATGG | CTCGGTGGCGTTGAGATTGTT |
| *β-actin* | GGCTGTATTCCCCTCCATCG | CCAGTTGGTAACAATGCCATGT |

**Table S2. Primers for quantitative RT-PCR.**
